# Reasons for performing a caesarean section in public hospitals in rural Bangladesh

**DOI:** 10.1186/1471-2393-14-130

**Published:** 2014-04-05

**Authors:** Mamuda Aminu, Bettina Utz, Abdul Halim, Nynke van den Broek

**Affiliations:** 1 Centre for Maternal and Newborn Health, Liverpool School of Tropical Medicine, Pembroke Place, Liverpool L3 5QA, UK; 2 Reproductive and Child Health Department, Centre for Injury Prevention and Research, Dhaka, Bangladesh

**Keywords:** Caesarean section, Indications, Bangladesh, Maternal mortality

## Abstract

**Background:**

It is estimated that 18.5 million Caesarean Sections (CS) are conducted annually worldwide and about one-third of them are done without medical indications and described as “unnecessary”. Although developed countries account for most of the rise in the trend of unnecessary CS, more studies report a similar trend in developing countries, putting a strain on existing but limited healthcare resources, jeopardizing families' financial security and presenting a barrier to equitable universal coverage. We examined indications for CS in public hospitals of one district in Bangladesh and explored factors influencing decision to perform the procedure.

**Methods:**

Retrospective review of case notes of 530 women who had CS in 5 public hospitals in Thakurgaon District of Bangladesh. Key Informant Interviews (KII) with 18 service providers to explore factors associated with the decision to perform a CS.

**Results:**

The commonest recorded indications for CS were: previous CS (29.4%), fetal distress (15.7%), cephalo-pelvic disproportion (10.2%), prolonged obstructed labor (8.3%) and post-term dates (7.0%). The majority (68%) of CS were performed as emergency; mainly during daytime working hours. Previous CS and “post-term dates” were common indications for elective CS with “post dates” – the commonest indication for CS in primiparous women. 16.0% of all CS were conducted for cases where alternative forms of care might have been more appropriate. Providers reported not using protocols and evidence based guidelines even though these are available. Pressure from patients and relatives to deliver by CS strongly influenced decision making. External agents from private hospitals receive a financial reward for every CS performed and are present in public hospitals to “lobby” for CS.

**Conclusion:**

Factors other than evidence based practice or the presence of a clear medical indication influence providers’ decision to perform both elective and emergency CS in public hospitals in Bangladesh.

## Background

An increase in the number of caesarean sections (CS) conducted for non-medical indications is an important contributor to the global rise in CS rates [1-3]. It is estimated that up to one-third of the 18.5 million annually performed caesarean sections worldwide are conducted for non-medical indications and have been described as “unnecessary” [4]. Sixty percent of the world’s births occur in low income countries; whereas, middle and high income countries account for only 37.5% of all births. To date, middle and high income countries contribute most to the global increase in CS rates [4].

Bangladesh is ranked as a low income country [5]. Despite its relatively low rate of skilled birth attendance (26.5%) [6], Bangladesh is nevertheless experiencing a rise in CS rates. Recently, the rate of caesarean deliveries has increased six-fold, from 2.7% in 2001 [6] to 17% in 2011 [7].

A rise in CS rates, however, is not necessarily associated with improvements in maternal and perinatal health indicators or quality of care [8]. Rather, it could increase the risk of maternal morbidity and mortality [9,10].

The International Federation of Gynecologists and Obstetricians (FIGO) issued a statement regarding the rising CS rates:

‘FIGO considers surgical intervention without a medical rationale to fall outside the bounds of best professional practice. Caesarean delivery should be undertaken only when indicated to enhance the well-being of mothers and babies and improve outcomes’ [11].

In contrast to the rise in CS in some settings, in many resource-poor countries, there are gaps in service provision with population based CS rates well below the UN recommended minimum of 5-15% [12,13].

Conducting “unnecessary” CS for women who do not really need this intervention could put a strain on existing and limited healthcare resources [14] as well as family financial security [15,16] and become a barrier to achieving universal and geographically equitable coverage with CS where this is medically indicated [4].

It is important, therefore, to study the rising trends in CS rates across lower and middle income countries (LMIC) and explore the reasons for this. An earlier study from Bangladesh (2007–8) of 400 CS conducted suggested that 12.5% had no clear medical indication recorded [17].

Standardisation of hospital record keeping systems including for CS and the monitoring of indications for CS is widely recommended.

We conducted this study to examine the reported indications for CS at all public hospitals in one district in Bangladesh and explored factors influencing the decision-making process of healthcare providers.

## Methods

This was a mixed method study using both quantitative and qualitative methodology. The study took place from July to December 2011 in the Thakurgaon District of Bangladesh, which consists of semi-urban and rural dwellings, with the majority of the population working as farmers. Women and children in the district benefitted from free healthcare under the Maternal and Newborn Health Initiative (MNHI) programme.

For the quantitative part of the study, case files of all patients from all 5 public hospitals in the district who had CS during the study period were included. In addition, we conducted key informant interviews (KII) with 18 healthcare providers purposively selected based up on their involvement in decision making regarding delivery via CS. Participants comprised of Obstetricians, Anesthetists, Medical Officers (MO) and Nurse-Midwives (NM).

We identified all patients who had CS from July to December 2011 from the operation theatre registers. It was possible to retrieve all (100%) of the case notes from the records office in each hospital. Information from the theatre register (Name, Date of birth, Date of CS) was cross-checked with patient files as well as admission ward registers. Information on patients’ demographic and pregnancy characteristics as well as the indication(s) for CS were extracted from the case notes.

Descriptive analysis was conducted using Epi Info 7 and SPSS Version 20. A topic guide was developed for use during the KIIs. All interviews were recorded, translated and transcribed. Qualitative data was analyzed using the thematic framework approach [18].

### Ethical approval

This study was approved by the Ethics Committee of the Liverpool School of Tropical Medicine and the National Research Ethics Committee of the Bangladesh Medical Research Council. Permission to conduct the study and use hospital data was also obtained from the District Civil Surgeon. Written, informed consent was obtained from each of the participants of the KIIs.

## Results

During the study period, 2,503 deliveries occurred in the five public hospitals. Of of these, 530 (21.2%) were caesarean sections (Table 1).

**Table 1:** Number of Cesarean Sections per six months and CS rate for five district hospitals in Bangladesh

**Health facility**	**Total deliveries**	**Number of CS**	**CS rate (%)**
Hospital A (Referral Hosp.)	1135	262	23.1
Hospital B	518	127	24.5
Hospital C	400	34	8.5
Hospital D	306	52	17.0
Hospital E	144	55	38.2
TOTAL	**2,503**	**530**	**21.2**

A total of 474 (89.4%) of all patients who had CS in these five public health facilities originated from within the Thakurgaon District. The remaining 56 patients (10.6%) were from neighboring districts. The mean age of women who had a CS was 23.5 years (SD = 4.2), less than half (45.5%) of all women who delivered by CS were primiparous and 35.1% of women had a previous CS performed (Table 2).

**Table 2:** Summary of patients’ demographic and pregnancy characteristics

	**Hospital A (n = 262)**	**Hospital B (n = 127)**	**Hospital C (n = 34)**	**Hospital D (n = 52)**	**Hospital E (n = 55)**	**Total (n = 530)**	**% of Total**
**Age of mother (years)**							
≤ 19	35	21	0	4	9	69	**13.0**
20 – 29	193	93	28	46	42	402	**75.8**
30 – 39	31	13	6	2	3	55	**10.4**
≥ 40	2	0	0	0	1	3	**0.6**
No information	1	0	0	0	0	1	**0.2**
**ANC attendance**							
Yes	0	121	24	28	55	228	**43.0**
No	0	6	1	0	0	7	**1.3**
No information	262	0	9	24	0	295	**55.7**
**Parity**							
1	118	50	17	27	29	241	**45.5**
2 – 4	143	77	16	25	26	287	**54.1**
≥ 5	1	0	1	0	0	2	**0.4**
**History of previous CS**							
Yes	83	63	13	17	10	186	**35.1**
No	170	64	21	35	44	334	**63.0**
No information	9	0	0	0	1	10	**1.9**
**Gestational age at the time of CS**							
Preterm	53	0	1	1	1	56	**10.6**
Term	136	106	24	43	33	342	**64.5**
Post dates	73	21	9	8	16	127	**24.0**
No information	0	0	0	0	5	5	**0.9**

### Indication for CS

Indication for CS was documented in 99.4% of all retrieved patient records (Table 3). The five commonest indications for CS were (in descending order): one or more previous CS, fetal distress, cephalo-pelvic disproportion, prolonged/obstructed labor and ‘post term dates’, together accounting for 70.6% of all CS conducted. Gestational age is generally estimated from the date of last menstrual period and/or using ultrasound. The five commonest indications for an emergency CS almost mirrored those for all CS and were: previous CS, fetal distress, cephalo-pelvic disproportion, prolonged/obstructed labor and rupture of membranes. For elective CS, the five commonest indications were different: previous CS, ‘post term dates’, poor obstetric history, hypertensive disorders and oligohydramnios.

**Table 3:** Recorded Indications for all CS, elective and emergency CS

**INDICATIONS**	ALL CS N = 530 (%)*	**ELECTIVE CS N = 164 (%)**	**EMERGENCY CS N = 360 (%)**
Previous CS	156 (29.4)	85 (51.8)	69 (19.2)
Fetal distress	83 (15.7)	0	82 (22.8)
Cephalo-pelvic disproportion	54 (10.2)	10 (6.8)	44 (12.2)
Prolonged/Obstructed labor	44 (8.3)	0	44 (12.2)
Post term dates	37 (7.0)	28 (4.1)	9 (2.5)
Hypertensive disorders	24 (4.5)	6 (3.7)	18 (5.0)
Rupture of membranes	22 (4.2)	0	22 (6.1)
Breech presentation	21 (4.0)	5 (3.0)	15 (4.2)
Failed induction	19 (3.6)	0	19 (5.3)
Oligohydramnios	15 (2.8)	6	9 (2.5)
Poor obstetric history	13 (2.5)	12 (7.3)	1 (0.3)
Malpresentation (excluding breech)	11 (2.1)	1 (0.6)	10 (2.8)
Ante-partum hemorrhage	6 (1.1)	0	6 (1.7)
Reduced fetal movements	6 (1.1)	2 (1.2)	4 (1.1)
Unfavorable cervix	5 (0.9)	2 (1.2)	3 (0.8)
Multiple gestation	3 (0.6)	2 (1.2)	1 (0.3)
Maternal distress	2 (0.4)	0	2 (0.6)
Older primipara	2 (0.4)	2 (1.2)	0
Rhesus incompatibility	1 (0.2)	1 (0.6)	0
Anemia	1 (0.2)	0	1 (0.3)
Recurrent urinary tract infection	1 (0.2)	1 (0.6)	0
Labor pain	1 (0.2)	0	1 (0.3)
No indication recorded	3 (0.6)	1 (0.6)	0

*of total for category.

Indications such as ‘post term dates’, rupture of the membranes, unfavorable cervix and labor pain make up about 12.3% of all CS. An additional 3.7% of all CS might be considered to have been done for non-medical indications (i.e. anemia, older primpara, maternal distress, recurrent urinary tract infections, poor obstetric history).

Of the 530 patients in this study, there were 360 emergency CS (67.9%) and 164 elective CS (30.9%). Information was not available for six (1.1%) women. Indication for CS for these six was previous CS (2), fetal distress (1), breech presentation (1) and no indication recorded (2).

Women who had CS for the first time (primary CS) constituted 63.0% (334/530) of all the patients in this study, while repeat CS accounted for 35.1% (186/530). Fetal distress, CPD, post term, obstructed labor, breech presentation, “rupture of membranes” and failed induction accounted for about half (49.4%) of the primary CS, while 83.4% of the repeat CS had previous CS as sole indication.

Primiparous women were significantly more likely to have an emergency CS (193/242) compared to multiparous women (168/288) (79.8% vs 58.3%). But, 20.2% of all CS in primiparous women were done as an elective CS (49/242). The most common indication for a CS in primparious women was post dates (19%) followed by CPD (9.3%) and fetal distress (8.4%).

**Figure 1:**
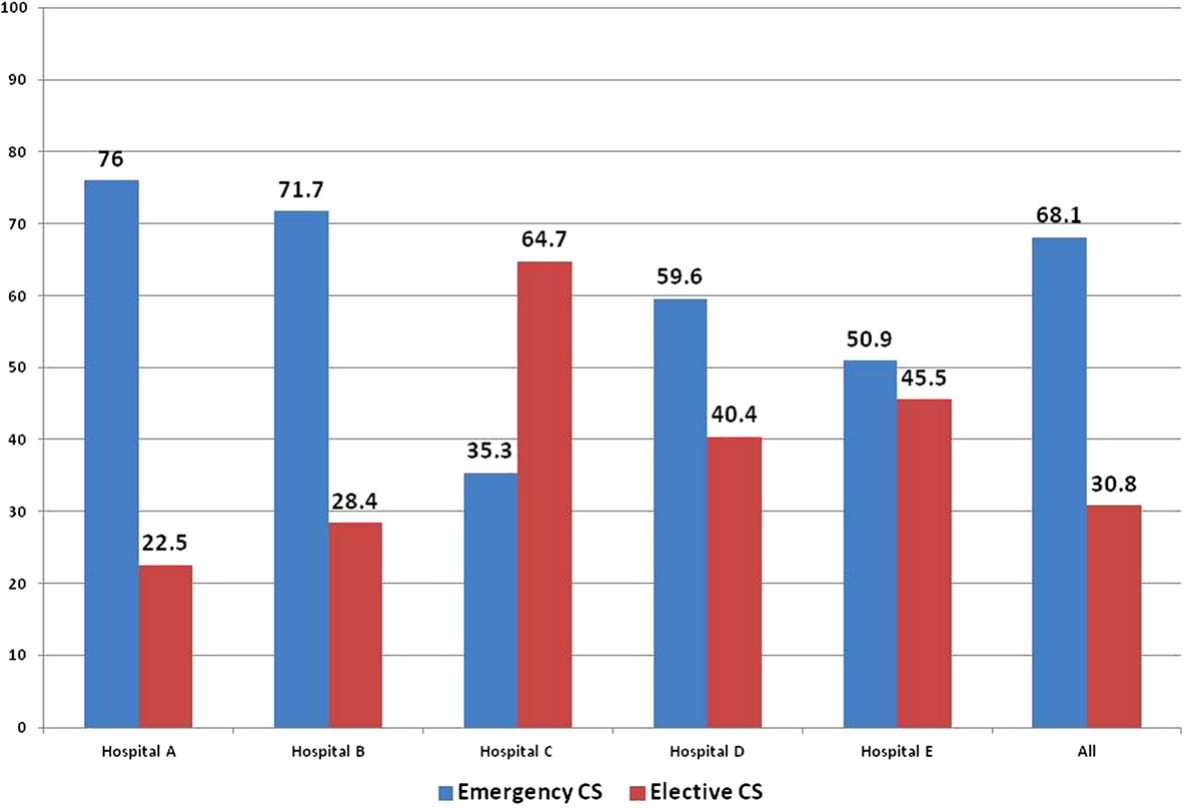
The proportion of CS that are done as emergency or elective by hospital.

Figure 1 compares the percentages of emergency and elective CS stratified by health facilities. With the exception of one hospital (Hospital E) which was not the main referral hospital most hospitals performed more emergency compared to elective CS.

**Figure 2:**
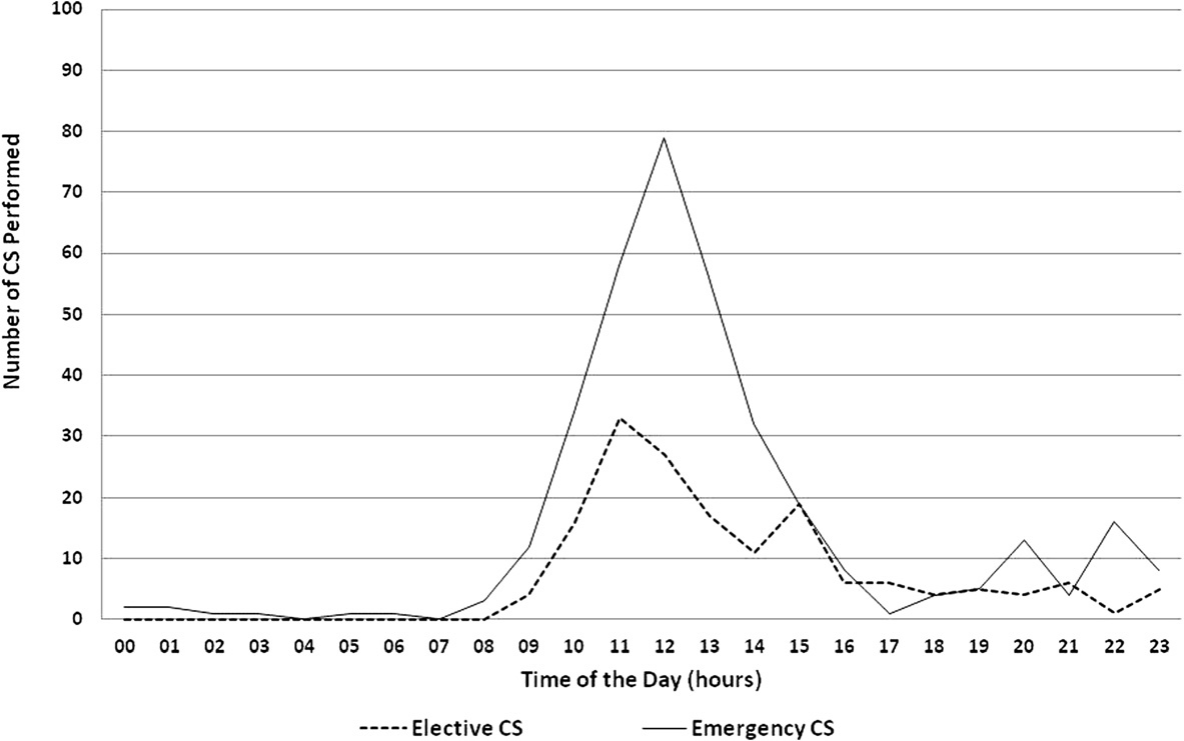
The number of CS performed in Bangladesh by time of day.

Overall, 76% (274/360) of emergency CS and 66% (108/164) of elective CS were performed during official working hours (between 08:00 hours to 14:00 hours). (Figure 2) Only one hospital contributed to the increase in emergency CS conducted between 20:00 and 22:00 hours.

### Factors influencing providers' decision to perform CS

Eighteen KII with healthcare providers were conducted. Factors that influence healthcare providers’ decision to perform CS were identified, with the following themes emerging.

Evidence based protocols and guidelines: The majority of the interviewees reported that protocols on emergency obstetric care (including some on CS) were available, but only a minority of providers adhere to these despite recognizing their benefits.

“We have protocols and guidelines… our nurses follow it, we follow it. But I think there are many big professors, big gynecologists (who) do not follow protocol. Their interest is CS. It happens in Bangladesh everywhere… If every sister follows protocol, if every doctor follows protocol, if every gynecologist follows protocol, then CS must reduce.” (MO #4)

For cases with previous CS, providers know a repeat CS is not mandatory but actual practice is different.

“Everywhere in Bangladesh, we (healthcare providers) recommend CS for every woman with previous CS. I asked about this during a training workshop… and I was told that only 25% to 30% of women with previous CS will need CS next time (pregnancy).” (MO #4)

Many providers also thought that the increasing rate of CS in Bangladesh was a major contributor to the reduction in maternal mortality and this might shape providers’ attitude towards performing a CS.

“Because we do more CS now, less number of women are dying…” (NM #4)

Pressure from families: Even when guidelines and protocols are available and followed, patients’ families frequently try to influence providers’ decision and ask for a CS even when there is no medical indication.

Providers reported that, there were several reasons, such as fear of loss of the baby or the mother, to explain why families may prefer CS instead of a normal delivery. Families put a lot of pressure on the providers to perform a CS often threatening that they would otherwise take the patient to another (often private) facility.

“Nowadays, they (patients) are too much conscious about their babies both in rural areas and in urban areas. Older patients or older family members sometimes think that they only need two children and they can do CS since they do not (want to) have future pregnancies. So, they sometimes tell us, ‘Do the caesarean section, do the caesarean section. If you do not want to do that, then you tell us, we’ll go somewhere else’.” (MO #3)

Even if medical personnel advise patients and relatives to opt for a normal delivery, this is not always respected. This is especially so when the patient comes from a more affluent background.

“Many patients desire CS, sometimes political leaders. But, we counsel them and advise them for a normal delivery. If they insist on CS (and we refuse), they leave the hospital.” (MO #1)

If providers do not respond to the demand of patients and families, the woman is often taken to another facility by the family where they can expect an operative delivery. This practice is supported by “agents” who are present in public hospitals and who facilitate transfer to neighboring private hospitals.

Effect of private healthcare: The majority of providers from public hospitals reported that agents often try and persuade patients to request a discharge from the public hospital so they can be taken to a private facility for a CS. Agents receive a financial reward from the private health care system in return. All providers interviewed were aware of this practice.

“In our hospital, there are many brokers. If CS is not done, they’ll contact the patient’s family to request for discharge to (a private) clinic. So, it is one of the important factors in decision in every place in Bangladesh.” (MO #4)

Fear of litigation: Other factors that promote a decision to perform a CS include fear of litigation and a lack of confidence in alternative interventions such as assisted vaginal delivery.

“The chance of litigation has increased. If the baby suffers asphyxia because of delay, they (parents) ask why CS was done late.” (Obstetrician #5)

“They (patients) are worried that baby’s condition will become worse if ventouse is used.” (MO #1)

Staff shortages: Some of the hospitals, particularly the rural hospitals, did not have the required minimum staff complement 24 hours a day (at least one cadre who could perform the CS and another to provide anesthesia). Such hospitals relied on visiting staff who had to finish work somewhere else first.

“Our own CS is more or less elective CS because after office hours we do not have all manpower and facilities available. So, we try to do CS in the afternoon. Electricity is also a factor, so we try to do (CS in) daylight. We don’t do it in the evening or night.” (Obstetrician #3)

## Discussion

This study has documented the indications for both elective and emergency CS conducted at district hospital level public hospitals in Bangladesh and explored how factors other than a medical reason for CS influence decision-making by health care providers.

Previous CS was the leading indication (29.4%) for CS in this study and was the main reason recorded for more than half of all elective CS. We estimated that the overall proportion of CS conducted without a clear medical indication could be considered to be 16.0% in this study. This could be higher if a more critical analysis of the indicators for CS were possible. For example, not all women who had had a previous CS may have required a repeat CS. CS for indications such as “post term dates”, unfavorable cervix and rupture of membranes could perhaps have been avoided if adequate guidelines and resources are in place for safe induction of labor or augmentation. It is a striking finding that the indication “post date” was recorded for almost 20% of all CS in primiparous young women. Other reasons for the “over-medicalization” of maternity care relate to a failure and inability to implement good quality evidence practices such as ensuring companionship during labor and delivery, a choice and range of drugs and methods to alleviate pain and assisted delivery where indicated using ventouse or forceps [19].

A study of 300 cases of CS in Pakistan conducted at a tertiary hospital noted 11.3% of these were elective CS and 88.7% were performed as an emergency CS [20]. Sultana et al. reported on 209 CS performed at district level in Pakistan with 11.9% done as an emergency and 82.4% as elective [21]. In Nepal at tertiary level, slow progress in labor, previous CS, fetal distress and breech presentation were the commonest indicators for CS [22]. Another study conducted in urban Bangladesh, reported fetal distress, pre-eclampsia and cervical dystocia as the commonest indications for CS [23].

Other studies from low and middle income countries suggest the proportion of CS performed without a clear reported medical indication might be higher. Maaloe et al. found that, of the 303 caesarean sections they reviewed in Tanzania, 25% were based on “inappropriate” indications and in an additional 38% of cases the indication was not clear [24]. Similarly, in this study, it is possible that some indications could be termed inappropriate e.g. for CS conducted for fetal distress if at birth there was no confirmation of fetal distress or incorrectly diagnosed CPD. Chu reported 20% of all CS in Taiwan may be performed without a clear indication [25].

A shortage of staff was given as the explanation for why most of the emergency CS, (76%) were conducted during daytime working hours. This is supported by the findings of Anwar et al. who reported that the unavailability of both an Obstetrician and Anesthetist when needed is often a reason why emergency obstetric care services including CS are not available 24/7 in rural areas of Bangladesh [26]. This is likely also to be a potential reason for the increase in the number of elective CS performed across the range of LMICs where the lack of medical staff is a problem. The inability to provide rapid emergency obstetric care (including CS) when this is needed outside of “normal working hours” is a real barrier to providing good quality, patient friendly, evidence based maternity care including CS for clear medical indication.

A range of factors other than adherence to evidence based guidelines and identification of a clear medical indication for a CS as mode of delivery influence providers’ decision to conduct CS in public hospitals at district level in Bangladesh.

Poor adherence with existing protocols highlights the need for clearer dissemination of guidelines for example via the professional associations. This includes re-orientation of healthcare providers on current evidence based obstetric care practices. Currently, the national guidelines for Bangladesh on delivery after previous CS follow WHO and FIGO guidelines both of which do not support a repeat CS unless there is clear indication for this. Healthcare providers are taught that “only 25-30% of women with a previous CS need a CS in the subsequent pregnancy”. However, as patient pressure and competing private interests also increasingly play an important role in the decision-making process, awareness should be raised among providers as well as among pregnant women and their families regarding the potential dangers associated with delivery by CS. Previous papers have highlighted the high “out of pocket” as well as regular cost of CS in LMIC including Bangladesh. However, this paper illustrates that despite this there is significant pressure from the private healthcare system encouraging women to deliver by CS even at district level and willingness and ability of rural families to pay for this. The introduction of demand-side financing mechanisms in Bangladesh has so far not been a key factor associated with increased CS rates [27,28].

## Conclusion

Performing a CS more frequently for cases where this is not really indicated constitutes an unnecessary strain on existing and already limited resources. Avoiding “unnecessary” CS would perhaps free resources and could contribute to making CS accessible to those who really need this intervention as part of the emergency obstetric and newborn care package that needs to be available 24/7 to manage potentially life threatening complications in both women and newborn babies.

### Limitations and further study

One of the limitations of this study is its focus on public hospitals only, all of which were located in the same district. This provides good information regarding what happens in public hospitals at district levels but we are aware that a substantial number of CS are increasingly being performed at private hospitals even in less urban settings in LMIC. Factors influencing decision making for CS reported in this study only reflect the views of healthcare providers. Patients’ views that would have highlighted other important aspects were not explored [29].

More research is needed to assess how health care providers can be supported to provide high quality patient friendly evidence based obstetric care and avoid over medicalization of care where this is not beneficial for women and their babies.

A recent Cochrane review highlighted that there is emerging evidence that guideline implementation, a mandatory second opinion and peer-review feedback could all lead to a reduction in CS cases. This requires further trials [3].

## Abbreviations

CS: Caesarean section; LMIC: Lower and middle income countries; MMR: Maternal mortality ratio; MO: Medical officer; NM: Nurse midwives.

## Competing interests

The authors declare that they have no known competing interests.

## Authors’ contributions

AH, BU & NvdB conceived of study which was designed by BU, MA & NvdB. The data was collected by BU & MA. All of the authors analysed the data and helped to draft and revise the manuscript. All authors read and approved the final manuscript.

## Pre-publication history

The pre-publication history for this paper can be accessed here:

http://www.biomedcentral.com/1471-2393/14/130/prepub
